# Dosimetry and Comparison between Different CT Protocols (Low Dose, Ultralow Dose, and Conventional CT) for Lung Nodules' Detection in a Phantom

**DOI:** 10.1155/2021/6667779

**Published:** 2021-01-22

**Authors:** Cleverson Alex Leitão, Gabriel Lucca de Oliveira Salvador, Priscilla Tazoniero, Danny Warszawiak, Cristian Saievicz, Rosangela Requi Jakubiak, Dante Luiz Escuissato

**Affiliations:** ^1^Department of Radiology, Universidade Federal do Paraná, Curitiba, Paraná, Brazil; ^2^Universidade Tecnológica Federal do Paraná, Curitiba, Paraná, Brazil

## Abstract

**Background:**

The effects of dose reduction in lung nodule detection need better understanding.

**Purpose:**

To compare the detection rate of simulated lung nodules in a chest phantom using different computed tomography protocols, low dose (LD), ultralow dose (ULD), and conventional (CCT), and to quantify their respective amount of radiation.

**Materials and Methods:**

A chest phantom containing 93 simulated lung nodules was scanned using five different protocols: ULD (80 kVp/30 mA), LD A (120 kVp/20 mA), LD B (100 kVp/30 mA), LD C (120 kVp/30 mA), and CCT (120 kVp/automatic mA). Four chest radiologists analyzed a selected image from each protocol and registered in diagrams the nodules they detected. Kruskal–Wallis and McNemar's tests were performed to determine the difference in nodule detection. Equivalent doses were estimated by placing thermoluminescent dosimeters on the surface and inside the phantom.

**Results:**

There was no significant difference in lung nodules' detection when comparing ULD and LD protocols (*p*=0.208 to *p*=1.000), but there was a significant difference when comparing each one of those against CCT (*p* < 0.001). The detection rate of nodules with CT attenuation values lower than −600 HU was also different when comparing all protocols against CCT (*p* < 0.001 to *p*=0.007). There was at least moderate agreement between observers in all protocols (*κ*-value >0.41). Equivalent dose values ranged from 0.5 to 9 mSv.

**Conclusion:**

There is no significant difference in simulated lung nodules' detection when comparing ULD and LD protocols, but both differ from CCT, especially when considering lower-attenuating nodules.

## 1. Introduction

Lung cancer represents the main cause of cancer-related deaths in the world [1]. Conventional chest radiographs used to be a screening tool for early diagnosis [2], but low-dose computed tomography (LDCT) proved to be superior [3]. The National Lung Screening Trial (NLST) was the first large multicentric study to show a reduction of 20% in lung cancer mortality in patients enrolled in a screening program using LDCT [4]. Nowadays, the American Cancer Society defends lung cancer screening using LDCT in smokers or former smokers who quit smoking in the last 15 years, aged between 55 and 74, with at least 30 pack-years smoking history [5].

The main issue regarding lung cancer screening is the systematic exposition of patients to ionizing radiation, which is potentially carcinogenic [6]. Patients submitted to annual LDCT have an additional risk of induced major cancers of 0.05% [7].

The pursuit for lower doses led to the creation of ultralow-dose (ULD) protocols, performed in modern CT equipment with iterative reconstruction [8] which expose the patient to amounts of X-rays as low as chest radiography [9].

This study aims to quantify the amount of radiation the patients are exposed to during a specific ultralow-dose protocol and different low-dose protocols and to determine how those doses impact the detection of simulated lung nodules in a phantom.

## 2. Materials and Methods

### 2.1. Phantom

The Alderson Rando radiotherapy phantom (RSD phantoms, Long Beach, USA) simulates a male patient (5 ft. 9 in. tall and weighing 162 lb.). It is composed of 2.5 cm thick slices, which contain pins that can be replaced by thermoluminescent dosimeter holders. It is molded of tissue-equivalent material, with lungs made from synthetic foam with a specific gravity of 0.30 g/cc. The pins in each slice have different CT Hounsfield values, which we used to simulate solid and ground-glass lung nodules.

The phantom contains a total of 105 simulated nodules, each one measuring 5 mm. Twelve of those were excluded from analysis due to their interface with the chest wall or mediastinum. The remaining 93 nodules were divided into columns and rows and named in accordance with their position. Three pinholes were left empty (positions E2, E11, and F9), so that they produced negative images (air density) that would help the participants count rows and columns.

We evaluated the mean attenuation coefficient in Hounsfield Units of each lung nodule in all the protocols by placing a 20 mm^2^ region of interest inside them, in order to verify if the ones with lower densities (ground-glass nodules) would have lower rates of detection in lower-dose protocols. The nodules attenuation coefficient ranged from −707 to −435 HU. The nodules were divided into three groups: density lower than −600 HU, density between −600 and −500 HU, and density higher than −500 HU. Thirty-two nodules (35%) had attenuation values lower than −600 HU, 48 (51%) between −600 and −500 HU, and 13 (14%) higher than −500 HU.

### 2.2. CT Protocols

All chest CT examinations were acquired using 256-slice multidetector CT (GE Revolution; General Electric Healthcare, Milwaukee, USA). We first used a usual high-resolution chest CT (CCT) protocol to obtain reference images: 0.5 s gantry rotation time, 120 kVp, 0.984 : 1 beam pitch, 40 mm table feed for gantry rotation, and *z*-axis tube current modulation.

To obtain LD and ULD acquisitions, the automatic tube current modulation was replaced by manually chosen values of mA, while the other parameters were maintained. For the ULD protocol, kVp was lowered to 80 and mA was set to 30. We decided to experiment with different low-dose protocols to verify how reductions in kV and mA affect the dose and nodule detection, since previous studies show that CT performed for other reasons, like pulmonary embolism, can be performed with lower doses and similar detection rates when those parameters are modified [10]. For LD protocol A, (lower mA) mA was lowered to 20 and, for LD protocol B (lower kVp), kVp was lowered to 100. LD protocol C (conventional LDCT) followed the AAPM recommendations for lung cancer screening [11].  Table 1 specifies the protocols used.

Images were reconstructed at 0.625 mm thickness, 512 × 512 matrix, using iterative reconstruction (ASiR-V™ General Electric Healthcare, Milwaukee, USA).

### 2.3. Image Evaluation

**Figure 1:**
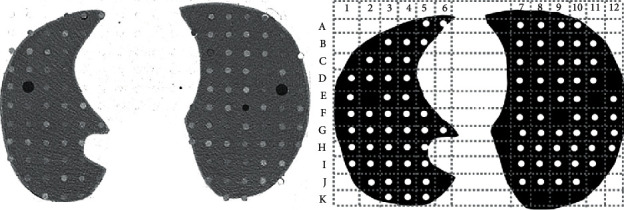
Example of the selected CT slice the radiologists analyzed and the printed diagram in which they registered the nodules they detected. Peripheral nodules such as A3, A4, and B2 were excluded from analysis because of their interface with the chest wall. Positions E2, E11, and F9 had no simulated nodules and served as reference to count columns and rows.

The resulting images were analyzed on a Picture Archiving and Communication System (CARESTREAM Vue PACs; Carestream Health, Inc., Rochester, USA) by four radiologists with at least 10 years of experience in chest radiology over the course of five weeks. The participants received a printed diagram in which the lung nodules were represented in columns and rows (Figure 1). Radiologists were asked to register in which locations they were confident enough to report the presence of a lung nodule. Images were displayed with a lung window setting, the same one used when the radiologists interpret clinical patient images (window level: −800/window width 1300). The radiologists were allowed to change the window settings as they pleased. Images were evaluated in a 3-megapixel, 500 cd/m^2^ maximum-luminance monitor (Barco Medical Displays, Duluth, USA).

### 2.4. Radiation Dose Assessment

Dosimetry was performed by placing lithium-fluoride thermoluminescent dosimeters (TLDs) over the surface of and inside the phantom (5 centimeters under the surface, at the level of the second row of nodules in the right lung), to estimate both entrance surface dose and dose delivered to the lung in each protocol. Since a single scan exposed the dosimeters to a very small amount of radiation, TLDs were scanned five times each and the mean dose for a single exposure was calculated. TLDs were previously calibrated with the aid of an ionizing chamber, being exposed to a specific amount of kV (80, 100, or 120) according to the protocol in which they would be used.

The mean dose absorbed by the dosimeters was converted to equivalent dose using the weighting factor (wR) value of 1 [12].

### 2.5. Statistical Analysis

Since nodules detection is a qualitative dataset (detection/no detection), thus, not normally distributed, we used nonparametric methods to estimate the significance interval. The comparisons of lung nodules detection with all five protocols were performed using the Kruskal–Wallis test [13]. Furthermore, McNemar's test was used in relation to the factor levels pairs to confirm which of the pairs were not similar, thereby causing a difference. The *p* value for statistical significance was *p* < 0.05.

In order to rate interobserver agreement, we calculated the *κ*-values for each pair of readers and for each pair of protocols (*κ*-value 0–0.2: poor; 0.21–0.4: fair; 0.41–0.6: moderate; 0.61–0.8: substantial; 0.8–1: almost perfect).

All statistical tests (Kruskal–Wallis, McNemar's, and the Kappa statistical analysis) were performed using MedCalc® software, version 19.1.3 (MedCalc software, Mariakerke, Belgium) [14].

## 3. Results

### 3.1. Dosimetry

The estimated equivalent entrance surface dose was 0.5 mSv for ULD acquisition and 9.0 mSv for conventional CT. The equivalent entrance dose for conventional LDCT was 1.8 mSv. The reductions made in kV and mA had a small impact over the equivalent dose. The estimated entrance dose for LDCT protocol A (lower mA) was 1.32 mSv and for LDCT protocol B (lower kV) was 1.34 mSv. Estimated equivalent dose to the lung ranged from 0.6 mSv (ULD) to 9.0 mSv (CCT).  Table 2 summarizes the equivalent dose in each protocol and their respective uncertainty values.

### 3.2. Nodule Detection

**Figure 2:**
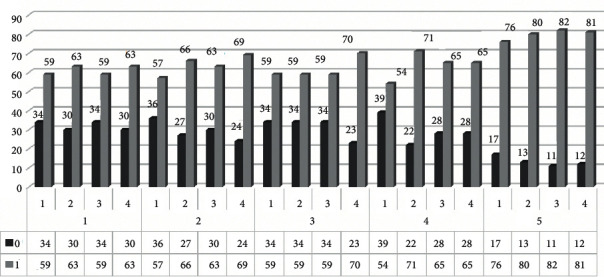
The number of nodules detected (gray columns, number 1) and missed (black columns, number 0) in each protocol (numbers 1 to 5, lower row) for every radiologist (numbers 1 to 4, upper row). Protocol 1: ULD, protocol 2: LD A, protocol 3: LD B, protocol 4: LD C, and protocol 5: CCT.

Nodule detection rate was 65.6% (±2.5% standard deviation) for ULD protocol, 68.5% (±5.5% standard deviation) for LD protocol A, 66.4% (±5.9% standard deviation) for LD protocol B, 68.5% (±7.6% standard deviation) for conventional LDCT, and 85.8% (±2.8% standard deviation) for conventional CT.  Figure 2 summarizes the number of nodules detected in each protocol by all radiologists.

For protocol 1 (ULDCT), 52 simulated nodules (56%) were detected by all radiologists, 8 nodules (10%) were detected by three radiologists, 4 nodules (4%) were detected by two radiologists, 4 nodules (4%) were detected by only one radiologist, and 25 nodules (26%) were missed by all radiologists.

For protocol 2 (LDCT—lower mA), 53 simulated nodules (57%) were detected by all radiologists, 9 nodules (10%) were detected by three radiologists, 6 nodules (6%) were detected by two radiologists, 4 nodules (4%) were detected by only one radiologist, and 21 nodules (23%) were missed by all radiologists.

For protocol 3 (LDCT—lower kV), 58 simulated nodules (62%) were detected by all radiologists, 3 nodules (3.5%) were detected by three radiologists, 3 nodules (3.5%) were detected by two radiologists, 10 nodules (11%) were detected by only one radiologist, and 19 nodules (20%) were missed by all radiologists.

For protocol 4 (conventional LDCT), 54 simulated nodules (58%) were detected by all radiologists, 8 nodules (9%) were detected by three radiologists, 7 nodules (7.5%) were detected by two radiologists, 6 nodules (6%) were detected by only one radiologist, and 18 nodules (19.5%) were missed by all radiologists.

For protocol 5 (CCT), 73 simulated nodules (79%) were detected by all radiologists, 5 nodules (5%) were detected by three radiologists, 2 nodules (2%) were detected by two radiologists, 8 nodules (9%) were detected by only one radiologist, and 5 nodules (5%) were missed by all radiologists.

The comparison between all five protocols revealed that protocols 1, 2, 3, and 4 showed no statistically significant difference in lung nodules detection rate (*p* > 0.05). However, each one of those protocols was statistically different from protocol 5 (*p* < 0.001).  Table 3 shows the *p* values obtained when comparing all protocols. Forty-three simulated nodules (46%) were detected by all radiologists in all protocols and only 3 nodules (3%) were not detected by any radiologist in any protocol. Six nodules (6%) were detected by one or more radiologists only in protocol 5. When comparing detection rates according to CT attenuation values, we detected that for lower-attenuating nodules (CT HU < −600 HU) every radiologist presented at least one low-dose or ultralow-dose protocol in which the detection rate was significantly different when compared to CCT (protocol 5). For radiologist 1, protocol 4 was different from 5 (*p*=0.024). For radiologist 2, protocols 1 and 3 were different from 5 (*p*=0.04). For radiologist 3, all protocols were different from 5 (*p*=0.007). For radiologist 4, protocols 1 and 4 were different from 5 (*p*=0.003 and *p*=0.007, resp.). There was no significant difference when comparing protocols for the detection rate of nodules with mean attenuation values from −600 to −500 HU (*p*=0.362) and higher than −500 HU (*p*=0.406).

*κ*-Values to evaluate interobserver agreement revealed that in all protocols there was at least moderate agreement (*κ*-value >0.41) between all radiologists, ranging from 0.522 to 1.000. When comparing the protocols (Table 4), the agreement between all ULD and LD was substantial (*κ*-values ranging from 0.617 to 0.766); however, their agreement levels when compared to CCT (protocol 5) were only fair or moderate (*κ*-values ranging from 0.398 to 0.502).

## 4. Discussion

Kim et al. [8] and Lee et al. [15] studied the same ULD protocol and reported effective doses of 0.31 and 0.29 mSv, respectively. As for the LD protocol, the mean effective dose in NLST was estimated in 1.5 mSv [16]. Depending on the values of kV and mA, literature shows different doses that range from 1.06 mSv to 2.7 mSv [8, 17]. Comparison between our results and others should be made carefully, due to different methodologies. Those studies report effective doses derived from DLP (dose-length product), a population dose metric that AAPM recommends not to be used to estimate dose or risk to an individual [11]. We decided to estimate dose using thermoluminescent dosimetry as an alternative that can be further reproduced by other authors.

Huber et al. [9] reported effective doses of 0.13 mSv using a ULD protocol, a value comparable to chest X-ray doses. The average effective dose for PA and lateral chest X-rays is approximately 0.16 mSv [18], what would make our protocol equivalent to about 3 X-rays. Again, this comparison is not entirely true due to different dosimetry approaches, but it still serves as an estimative of how CT can be performed with impressively low doses.

When trying to reduce patient exposure, ULDCT poses an interesting option, since it is possible to obtain considerably lower doses with similar nodule detection rate. When performing ULDCT is not an option, lowering kV and mA can reduce the equivalent dose without compromising image quality.

Literature shows conflicting results on the effects of dose reduction over lung nodules detection. Huber et al. [9] reported nodules detection rates of 93.3% in ULDCTs, compared to 95.5% in standard dose CTs (no significant difference). That study used a different anthropomorphic phantom, with different lung nodules sizes and attenuation values (−630 HU and 100 HU), which can partly explain the different results we obtained. Other studies show that low-dose images can reduce the detectability of peripheral lung nodules [19] and result in differences in nodules volumetry [20].

When evaluating interobserver agreement, it has been previously reported that even NLST participants presented substantial variability in false-positive rates [21]. Studies have shown that LDCT could result in poor accordance, with *κ*-values as low as 0.120 [22]. However, our degree of agreement is closer to the ones presented in more recent studies (kappa as high as 0.848 for 80 kV LDCT) [20]. The lowest *κ*-value for low-dose protocols in this study was 0.600, which still represents moderate agreement. The comparison of the performance of the radiologists between ULD and LD protocols revealed substantial agreement (kappa ranging from 0.605 to 0.766), indicating that although there was a small degree of heterogeneity, the final performance of the observers in different protocols was comparable, as shown in previous research [23].

Considering the effects of radiation dose over the detection of ground-glass nodules is also important. Literature shows that lepidic-growth adenocarcinomas tend to show CT attenuation values between −651 and −447 HU [24]. In this study, all 93 simulated lung nodules had attenuation values within this range. Although ULD and all three LD protocols had similar rates of detection for nodules with attenuation values lower than −600 HU, there was a significant difference when comparing the detection of those nodules with conventional dose CT (CCT). Funama et al. found that images obtained with lower milliampere settings (21 and 45 mA) can reduce the detection of simulated ground-glass lung nodules with attenuation coefficients of −650 HU in a phantom [25]. Our study reproduces this finding, showing that special attention should be given to lower-attenuating nodules, as scans performed with LD and ULD protocols can potentially miss small lepidic-growth adenocarcinomas.

Even though this study detected significant differences in lung nodules detection when comparing ULD and LDCT to conventional CT, the risk of radiation-induced cancer still has to be taken into account for all lung cancer screening programs. The dose reduction in LDCT can be responsible for reducing the risk of carcinogenesis from 8.6 to 0.35 per 100000 cases [26]. The “ALARA” (as low as reasonably achievable) principle of dose containment is still where the main efforts for lung cancer screening are driven [27].

This study has limitations: the main one being the use of a phantom instead of real patients. The methodology we chose for this study, in which five different protocols were repeated five times, would expose human participants to high levels of radiation, what would be ethically questionable. Our phantom simulates a 162 lb (73 kg) male, what limits the extrapolation of these results to different populations since different body types can have different qualities of images when scanned with the exact same protocols. There need to be further clinical studies in order to assure our results are reproductible in human patients and in different populations. Furthermore, all the analysis in this study were performed by radiologists, in contrast to many other similar ones that use computer aided detection software capable of reducing detection variability in lung nodules in different protocols [28]. Automatic tools have already proved to be efficient in detecting potentially malignant microcalcifications in digital mammograms [29] and are progressively being implemented for malignant nodules detection in CT scans [30], which can help to overcome human-analysis limitations potentially identifiable in this study.

In conclusion, it is possible to perform ULDCT exposing patients to less than a third of the equivalent dose seen in conventional LDCT and less than a tenth of conventional CT. There is no significant difference in lung nodules detection rates when comparing images obtained with ULDCT and LDCT. However, the detection rate for ULDCT and LDCT protocols differs from conventional CT, especially for lung nodules with lower density (CT Hounsfield units <−600 HU). These differences should be taken into account when selecting a protocol for the implementation of lung cancer screening programs.

## Figures and Tables

**Table 1:** Protocol specifications.

	ULD	LD (lower mA)	LD (lower kV)	Conventional LDCT	HRCT
Gantry rotation time (s)	0.5	0.5	0.5	0.5	0.5
Beam collimation (mm)	40	40	40	40	40
Pitch	0.984	0.984	0.984	0.984	0.984
kV	80	120	100	120	120
mA	30	20	30	30	Auto/smart mA

**Table 2:** Estimated equivalent doses.

Protocol	kV	mA	Equivalent entrance skin dose/uncertainty (mSv)	Equivalent dose to the lung/uncertainty (mSv)
Ultralow dose	80	30	0.5/0.06	0.6/0.06
Low dose (lower mA)	120	20	1.32/0.26	1.32/0.12
Low dose (lower kV)	100	30	1.34/0.3	1.5/0.3
Conventional low dose	120	30	1.8/0.36	2.0/0.16
Conventional HRCT	120	Automatic	9.0/0.8	9.0/0.58

**Table 3:** Comparison of the number of nodules detected by all radiologists for every pair of protocols using McNemar's test.

Protocols	1/2	1/3	1/4	1/5	2/3	2/4	2/5	3/4	3/5	4/5
Nodules detected	244/255	244/247	244/255	244/319	255/247	255/255	255/319	247/255	247/319	255/319
*p* value	0.208	0.749	0.215	<0.001	0.302	1.000	<0.001	0.322	<0.001	<0.001

**Table 4:** Comparison of nodule detection rates between pairs of protocols (kappa values).

	Protocol 1	Protocol 2	Protocol 3	Protocol 4	Protocol 5
Protocol 1	—	0.617	0.766	0.605	0.398
Protocol 2	0.617	—	0.718	0.688	0.385
Protocol 3	0.766	0.718	—	0.694	0.494
Protocol 4	0.605	0.688	0.694	—	0.502
Protocol 5	0.398	0.385	0.494	0.502	—

## Data Availability

No data were used to support this study.
